# Where are we in the diagnosis and treatment of hirsutism? A narrative review

**DOI:** 10.1007/s11154-026-10057-2

**Published:** 2026-06-03

**Authors:** Mert Yesiladali, Kursad Unluhizarci, Erkut Attar, Fahrettin Kelestimur

**Affiliations:** 1 Faculty of Medicine, Obstetrics & Gynecology Department, Reproductive Endocrinology Unit, Yeditepe University, Istanbul, Turkey; 2 Faculty of Medicine, Endocrinology Department, Erciyes University, Kayseri, Turkey; 3 Faculty of Medicine, Endocrinology Department, Yeditepe University, Istanbul, Turkey

**Keywords:** Hirsutism, Hyperandrogenism, Polycystic ovary syndrome, Androgen excess, Hair follicle physiology

## Abstract

**Background:**

Hirsutism is a prevalent androgen-dependent condition that significantly affects women’s physical and psychological health. Despite decades of study, major gaps persist in understanding local androgen metabolism, the role of insulin resistance, and individualized treatment responses. This review aims to provide a contemporary update that integrates recent molecular, diagnostic, and therapeutic advances.

**Objective:**

To critically appraise the current understanding of hirsutism, with an emphasis on: (1) emerging concepts in local androgen action and follicular sensitivity, (2) evolving assessment methods, (3) updated pharmacologic and mechanical treatment options aligned with current endocrine and dermatologic evidence.

**Search methods:**

A narrative review of the literature was conducted in PubMed, Scopus, and Web of Science for studies published between January 2000 and September 2025, using the terms: “hirsutism,” “hyperandrogenism,” “androgen excess,” “idiopathic hirsutism,” “PCOS,” “5α-reductase,” “antiandrogens,” “oral contraceptives,” “laser hair removal,” and “insulin resistance.”

Inclusion criteria: human studies, English language, adult females, and peer-reviewed original or review articles.

**Wider implications:**

An integrated understanding of systemic and local androgenic activity is transforming the management of hirsutism from a purely cosmetic issue to a complex endocrine disorder with metabolic and psychological dimensions. This review proposes a personalized, multidisciplinary framework that bridges endocrinology, gynecology, and dermatology. Future directions should focus on elucidating local androgen metabolism, tissue-specific enzyme activity, and the role of insulin resistance in idiopathic cases to refine both diagnostic precision and treatment efficacy.

## Introduction

Hirsutism is not a disease per se, rather it is an androgen dependent symptom, similar to androgenetic alopecia, acne and seborrhea. Hirsutism, characterized by excessive growth of terminal hair in a male-like distribution pattern in women, is a common clinical condition that can significantly impact quality of life and psychological well-being. The complex pathophysiology of hirsutism involves various hormonal, genetic, and environmental factors, with androgens playing a central role. The manifestations may significantly affect self-esteem, body image, and emotional well-being. Women with hirsutism may experience social stigmatization and anxiety, which can lead to depression or other mental health issues [1]. Moreover, hirsutism may be an initial sign and symptom of an ovarian or adrenal androgen secreting tumor. Therefore, proper diagnosis, evaluation, and management of hirsutism are crucial not only for addressing the physical manifestations of the condition but also for improving the psychological well-being of affected individuals as well as early management of life-threatening disorders such as adrenal carcinoma.

This review aims to summarize the current state of knowledge regarding the diagnosis and treatment of hirsutism, evaluate recent advances and emerging therapies and identify areas where further research is needed to improve patient care and outcomes. Given the breadth of the topic and the heterogeneity of available evidence, this review was conducted as a narrative review with the aim of providing a clinically oriented synthesis of current knowledge.

## Definition of hirsutism

Hirsutism is defined as a clinical condition characterized by the excessive growth of terminal hair in women with a male-like distribution pattern. Terminal hair is coarse, pigmented, and longer compared to the fine, non-pigmented vellus hair that typically covers most of the body [2]. In women with hirsutism, hair growth commonly occurs on the mid-line areas of the body such as face, chest, abdomen, and back [3]. Hirsutism can result from elevated androgen levels, increased sensitivity to androgens, or a combination of both factors [4].

While hirsutism is primarily an aesthetic concern, it can also be an indicator of underlying hormonal imbalances or medical conditions, such as polycystic ovary syndrome (PCOS) or adrenal disorders [5]. In some cases, hirsutism may be idiopathic, meaning that the patient has normal serum androgen levels and no specific cause can be identified [6]. The severity of hirsutism can be assessed using various scoring systems, with the modified Ferriman-Gallwey (mFG) score being the most widely used [7]. In most cases the degree and distribution of hirsutism do not suggest any specific cause of androgen excess disorder.

## Epidemiology of hirsutism

The prevalence of hirsutism varies depending on the population studied, the diagnostic criteria used, and the assessment methods. In general, hirsutism is estimated to affect between 5 and 15% of women of reproductive age worldwide [6]. However, these percentages may not accurately reflect the true prevalence of the condition for all nations, as cultural/ethnic factors and individual perceptions of hair growth may influence reporting and diagnosis.Racial and ethnic differences play an important role in the prevalence of hirsutism worldwide. For example, hirsutism is more common in some ethnic groups including Mediterranean, Middle Eastern, European, and South Asian ethnic groups [8]. On the other hand, East Asian populations exhibit lower hirsutism rates even among PCOS patients, emphasizing an important ethnic and genetic factor in the expression of hirsutism [9]. Hence, different Ferriman-Gallwey threshold scores have been proposed among nations. Recognizing the variation among different ethnicities is essential for healthcare providers to employ tailored diagnostic criteria to women with hirsutism.

## The pathophysiology of hirsutism

Hirsutism is the result of a complex interaction between circulating and local androgen concentration and the sensitivity of hair follicles to androgens. Basic knowledge on the hair types in the body as well as the anatomy and physiology of the hair follicle is helpful in understanding the pathophysiology of hirsutism. Broadly, human hair can be classified into three main types: lanugo, vellus, and terminal hair, each characterized by unique morphological features and locations.

### Lanugo Hair

Lanugo is the first type of hair produced by fetal hair follicles during gestation. This fine, soft, and unpigmented hair begins to grow at about 12 weeks of gestation and is usually shed and replaced by vellus or terminal hair by the 36th week or shortly after birth [10].

### Vellus hair

Vellus hair is a short, fine, light-colored and barely noticeable hair type. It covers most of the human body except areas like the palms, soles, lips, and certain external genital areas. Vellus hair is less than 2 mm in length and does not have a medulla, the central core found in terminal hairs. under the influence of hormonal changes, vellus hairs in some areas transform into terminal hair during puberty [10, 11].

### Terminal hair

Terminal hair is thick, long, and pigmented, found on the scalp, eyebrows, eyelashes, and, after puberty, in areas such as the axilla and pubic region. In men, terminal hair also grows on the face, chest, and limbs. Hormones, especially androgens, are responsible for the growth of terminal hair, accounting for sex-related and interindividual differences in hair thickness and distribution [12].

### Hair follicle structure

The hair, along with a connected sebaceous gland and arrector pili muscle, makes up the structural, or pilosebaceous unit of a hair follicle. Hair follicles that give rise to terminal hairs can reach deep into the dermis and occasionally into the subcutis. In the meantime, vellus hair-producing follicles are limited to the upper reticular dermis.

There are two parts of a hair follicle: the upper and bottom sections. The bulb and suprabulbar region are the terms used to describe the lower segment, while the infundibulum and isthmus represent the upper segment [13]. The lower segment of hair follicle includes the bulb, which has the follicular matrix near the dermal papilla. The matrix, which has the fastest rate of mitosis of any organ, interacts with the papilla. The medulla is the name for the inner core of the hair shaft. The cortex, which comprises most of the hair, surrounds medulla. 

### Growth cycle of a hair follicle

Hair follicles undergo consecutive cycles of growth (anagen), regression (catagen) and rest (telogen) phases; thus, body hair regrowth is sustained. The hair shaft and the inner root sheath are produced by rapidly multiplying progenitor cells in the bulb during anagen phase. The length of the hair cycle in various body parts is determined by the duration of the anagen phase [14]. The anagen phase of hair follicle depends on body location; while it is longest in the scalp (two to six years), the anagen phase of facial hair is nearly four months. Therefore, it takes nearly six months for medical treatments in this area to take effect, and a significant effect can be observed around nine months [15].

### Androgen effect on pilosebaceous unit

Androgens, including testosterone and dihydrotestosterone (DHT), play a significant role in regulating hair growth cycle, particularly affecting the anagen (growth) phase. These hormones interact with hair follicles through androgen receptors, which are found in the dermal papilla cells of the follicles. Dehydroepiandrosterone (DHEA), dehydroepiandrosterone sulfate (DHEAS), and androstenedione are considered as pro-hormones because they either do not bind to androgen receptors or exhibit a very weak binding affinity [16]. Hair follicles that produce vellus-type hairs can be induced by androgens to produce terminal hairs.

**Fig. 1:**
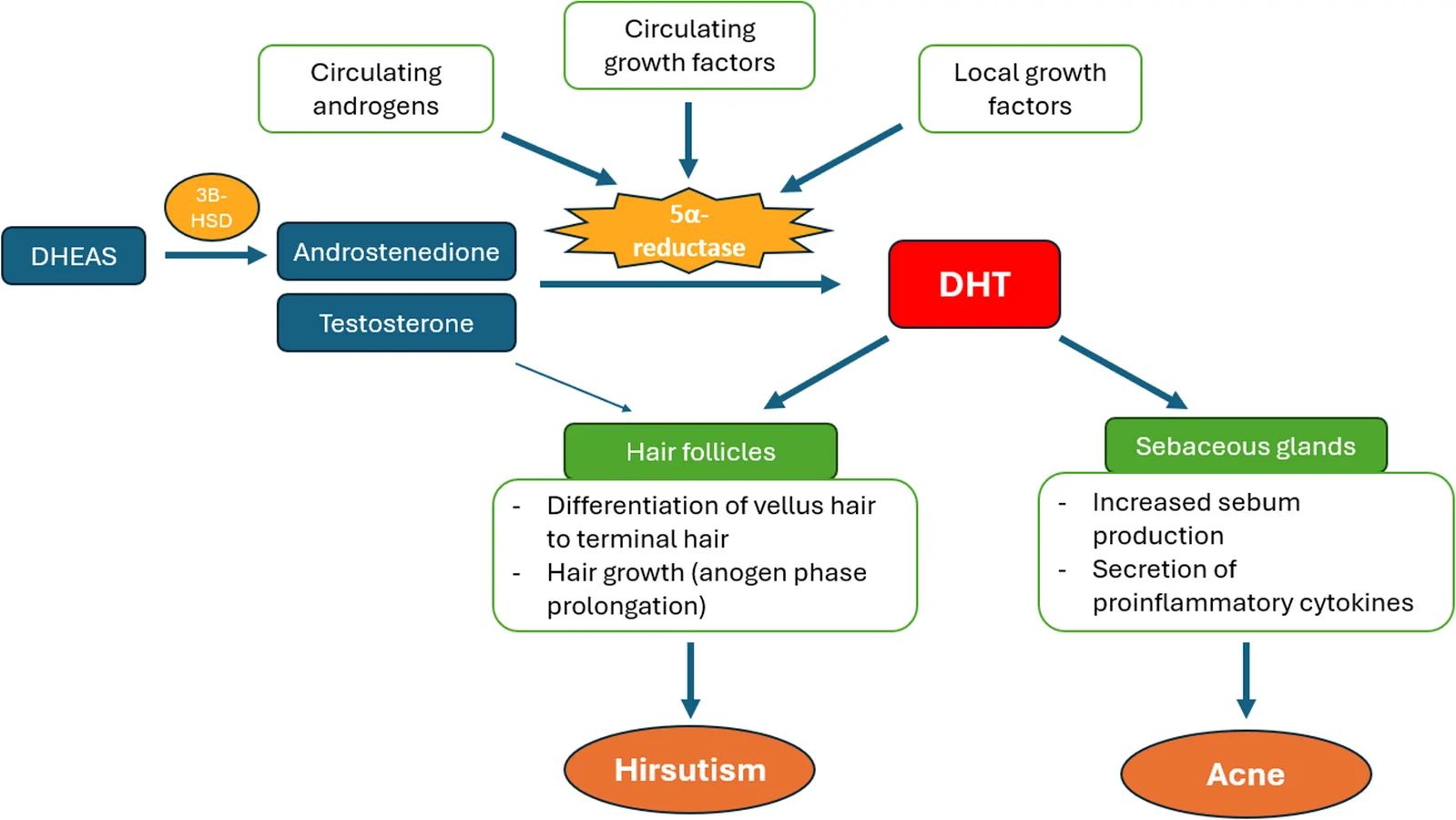
The androgen effects on pilosebaceous unit (3β-HSD: 3β-hydroxysteroid dehydrogenase, DHT: dihydrotestosterone)

Although testosterone also has direct effect, DHT is the primary hormone that affects hair follicles. The main source of DHT is peripheral non-hepatic 5α-reduction of testosterone [17]. The distribution and activity of 5α-reductase are affected by androgens and several other factors (Fig. 1 ). It has been shown that activity of this enzyme is influenced by both circulating (such as insulin-like factor-I, activin A, and inhibin A) and local growth factors (such as transforming growth factor-β and epidermal growth factor) leading to increased hair growth and activation of sebaceous glands resulting in acne formation [18, 19].

The 5α-reductase content and androgen metabolizing capacity of body hairs vary significantly among different body locations and persons [6]. In addition, the expression of the androgen receptor in the pilosebaceous unit is another crucial factor that determine the final androgen effect on hair [20]. The androgen receptor gene polymorphisms also affect the receptor activity [21]. Alterations in gene expression levels of local 5α-reductase and aromatase in women with idiopathic hirsutism have been reported previously indicating the importance of local androgen synthesis/metabolism [22]. These variations in hair follicle physiology among people constitute a reasonable explanation for absence of a direct proportion between circulating serum androgen levels and hirsutism severity. Knowledge that androgens can affect a hair follicle regardless of their blood levels is important to comprehend hirsutism, particularly idiopathic hirsutism (IH).

Because of their effect on the whole pilosebaceous unit, androgens also influence sebaceous glands and contribute to oily skin and the acne formation. It has been demonstrated that DHT is involved in both the synthesis of sebum and the secretion of proinflammatory cytokines by sebocytes [23]. Sebaceous glands from acne-prone areas, such as face skin, exhibit a stronger reductive activity of 17β-hydroxysteroid dehydrogenase (17β-HSD) indicative of a higher testosterone production, compared to non-acne-prone areas [24]. Specifically, the enzymes 17β-HSD 3 and 5 are particularly expressed at sebaceous glands found in the skin of the face. These enzymes catalyze the conversion of androstenedione to testosterone [25]. Figure 1 represents an illustration of the androgen effects on pilosebaceous unit.

## Assessment of a patient with hirsutism

Since it is usually a symptom of an underlying pathology, it is important to make a thorough assessment for hyperandrogenism. The clinical evaluation and diagnostic approach to a patient with hirsutism involve a detailed history, physical examination, selective laboratory testing and radiological imaging when necessary. The medical history should include the presence of menstrual irregularity, time of onset and progression rate of symptoms, family history, medication history and other common findings of hyperandrogenism. Depression is common in these patients due to social stigmatization; therefore it is important to question depression symptoms and refer to psychiatry if depression is suspected.

Physical examination includes assessment of severity of hirsutism, other signs of hyperandrogenism (acne, hair loss, seborrhea), signs of virilization (clitoromegaly, male pattern alopecia, deepening of voice), and signs of endocrine abnormalities including Cushing's syndrome, hyperprolactinemia (galactorrhea), acromegaly and thyroid disorders [15, 26, 27]. A general physical examination including body mass index (BMI) and blood pressure will aid in the diagnosis of a possible underlying condition. A detailed skin visualization is crucial and signs like acne, striae, or acanthosis nigricans should be noted [28].

There are some objective and subjective methods for the evaluation of hirsutism. Objective methods such as weighing of plucked hair, microscopic measurements or photographic evaluations are costly and not practical for daily use. Among the subjective methods, which are rely on visually scoring of terminal hairs, mFG scoring system has become the most preferred one. The severity of hirsutism is most commonly assessed using the mFG scoring system which includes hair density in nine body areas: upper lip, chin, chest, upper back, lower back, upper abdomen, lower abdomen, upper arms, thighs [29, 30]. mFG threshold scores according to ethnic origin are as follows: United States and United Kingdom black or white women, ≥ 8 [16]; Mediterranean, Hispanic, and Middle Eastern women, ≥ 9 to 10 [16]; South American women, ≥ 6 [31]; and Asian women, a range of ≥ 2 for Han Chinese women to ≥ 7 for Southern Chinese women [32, 33]. A structured clinical assessment is essential, as the diagnostic yield depends on a careful integration of history, examination, and selective laboratory testing. Table 1 summarizes the recommended evaluation framework, highlighting that some of the laboratory testing should be tailored to clinical findings rather than applied uniformly to all patients. For instance, dexamethasone suppression testing or adrenal/ovarian imaging is reserved for cases with rapid progression or signs of virilization, whereas insulin resistance assessment is increasingly relevant across the broader patient population.

**Table 1:** Assessment of a patient with hirsutism

Patient History	- Menstrual cycle - Time of onset and progression rate of symptoms - Family history - Medication history - Other common findings of hyperandrogenism (acne, hair loss, virilization) - Depression assessment
Physical Examination	- mFG score - BMI, blood pressure - Signs of virilization (clitoromegaly, male pattern alopecia, deepening of voice) - Signs of hyperandrogenism (acne, thinning and loss of scalp hair) - Acanthosis Nigricans - Signs of endocrine disorders (Cushing's syndrome, hyperprolactinemia, thyroid dysfunction, acromegaly)
Laboratory Assessment	- Total and free testosterone - SHBG - DHEA-S - Androstenedione - Free Androgen Index (FAI) = (Total testosterone / SHBG) x 100 - 17-OHP (17-hydroxyprogesterone) - 11-deoxycortisol (in populations where 11 Beta-hydroxylase deficiency is common) - Prolactin - Midluteal progesterone - Insulin / HOMA-IR - HbA1c, OGTT - Anti-Mullerian Hormone - TSH, fT4 - Oligomenorrhea patients: Prolactin, TSH - Ovarian USG - If signs of Cushing’s syndrome present: Dexamethasone suppression test - Rapid progression and aggressive symptoms: Adrenal/Ovarian CT or MRI - If required: IGF-1 and GH response to OGTT

Hirsutism should be differentiated from hypertrichosis which is characterized by increased vellus-type hair growth all over the body and does not reflect hyperandrogenism. It may be associated with hypothyroidism, anorexia nervosa or to some drugs such as diazoxide, cyclosporin and rarely it may be seen as a paraneoplastic syndrome which is called hypertrichosis lanuginose [34]. In some patients hypertrichosis and hirsutism coexists and in these patients the clinical expression of hirsutism is more severe than real hirsutism score.

Laboratory testing enables distinguishing hyperandrogenic hirsutism from idiopathic hirsutism and aids to make differential diagnosis. The most important issue is excluding ovarian or adrenal tumor as a cause of hirsutism. Recommendations for laboratory testing continue to evolve with ongoing research, however most guidelines support measuring one or more serum androgens in all patients with hirsutism. The consensus statement and guideline on diagnosis and management of hirsutism published by the Androgen Excess and Polycystic Ovary Syndrome Society in 2011 recommends laboratory testing of at least one serum androgen in every patient with hirsutism before starting any treatment [16]. Hormonal evaluation of the patient should be done in the follicular phase of menstrual cycle. In oligomenorrheic patients these investigations may be done after establishing low serum progesterone values indicating follicular phase of the cycle.

Of all the circulating androgens, assessment of free testosterone levels are emphasized to be much more sensitive than the measurement of total testosterone [35]. The value of measuring serum androstenedione and DHEAS levels in patients with hirsutism is unclear but may be helpful in detecting ∼10% of hyperandrogenic patients [35]. These recommendations slightly differ from those of the 2018 Endocrine Society Clinical Guideline, which encourages initially testing total testosterone only [15]. In patients who have normal total testosterone levels but have moderate/severe hyperandrogenism, the guideline recommends obtaining free testosterone levels. The Society of Obstetricians and Gynaecologists of Canada (SOGC) 2023 hirsutism guideline recommends measuring total testosterone, DHEA-S, and SHBG in all patients with an abnormal mFG score [36]. It is widely recommended that all patients with hyperandrogenic hirsutism be tested for 17-OHP to rule out nonclassic congenital adrenal hyperplasia (NCAH) [37–39]. On the other hand, 11 beta hydroxylase deficiency should not be overlooked in certain populations as a cause of NCAH [40].

For the diagnosis of hyperandrogenemia, measurement of free testosterone level is more sensitive than measurement of total testosterone [35]. However, accurately measuring free testosterone with current and widely used laboratory measurement techniques is challenging. Equilibrium dialysis procedures are the optimal method for measuring free testosterone. Direct analog radioimmunoassay methods are not recommended for free testosterone measurement [41]. Calculating free androgen index (FAI), which is the percentage ratio of total testosterone to SHBG concentration, is a more reliable method in clinical settings where direct measurement of free testosterone may be problematic [42].

When symptoms and signs suggesting Cushing’s syndrome are present, a dexamethasone suppression test should be performed [43]. Measurement of serum prolactin is necessary if galactorrhea or irregular menstruation are present, as this is an uncommon cause of hirsutism. It is recommended that all patients with oligo-amenorrhea should get a b-HCG test [36]. A pelvic ultrasonography may confirm the presence of polycystic ovarian morphology or an androgen-secreting ovarian neoplasm. In patients with hirsutism, particularly those with PCOS, body composition holds significant importance and body fat distribution may be predictive of metabolic profile [44]. Therefore, conducting anthropometric measurements under the guidance of a dietitian during the evaluation phase is valuable. Insulin resistance is another common condition observed in these women. Although screening for insulin resistance is widely recommended for women with obesity-related hirsutism, studies have demonstrated that insulin resistance is common among women with hirsutism independent of their body mass index (BMI) [45]. Therefore, it can be considered beneficial to assess insulin resistance in all women presenting with hirsutism.

## Causes of hirsutism

**Fig. 2:**
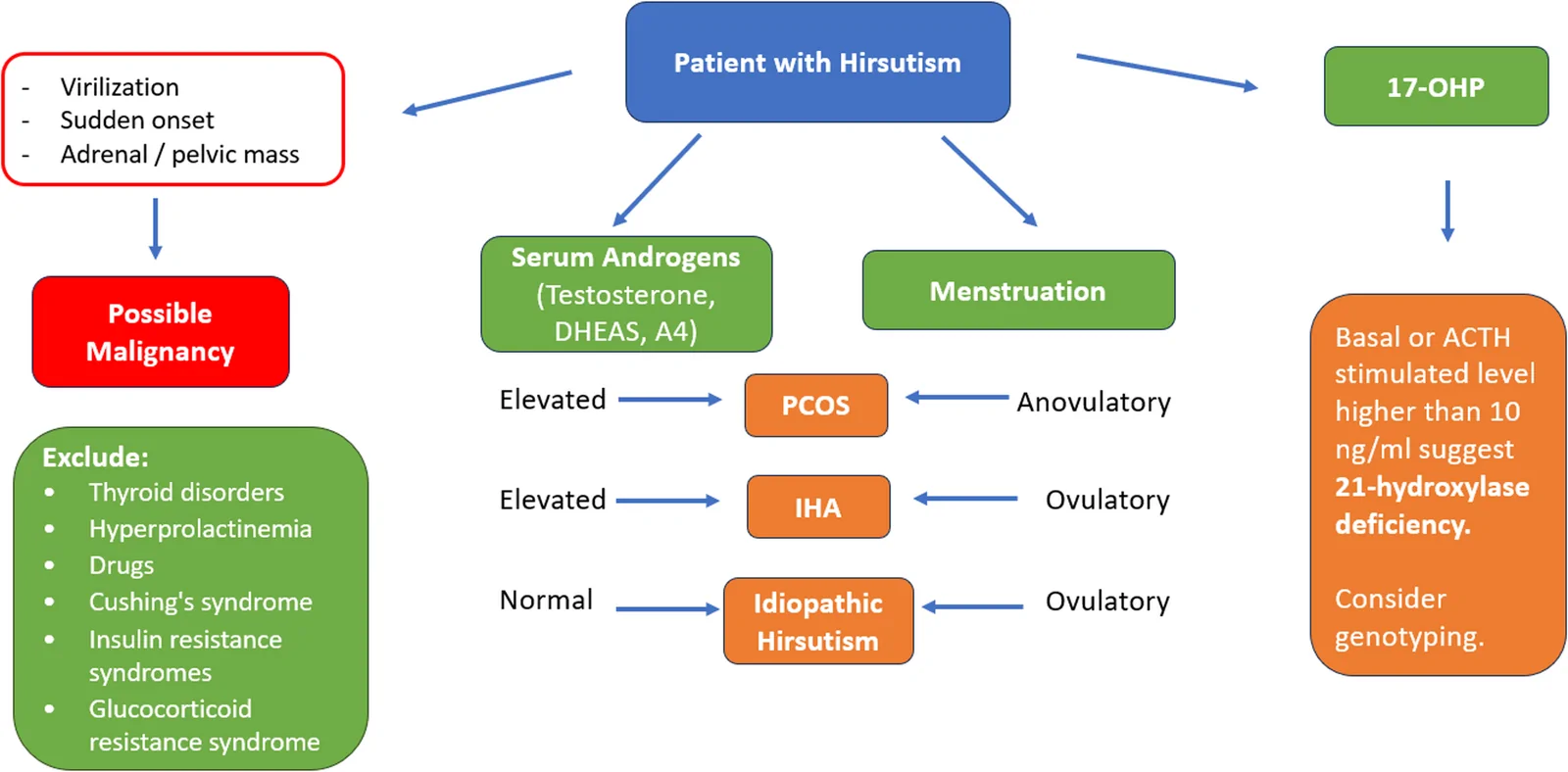
Diagnostic Algorithm for Hirsutism (A4: androstenedione, IHA: idiopathic hyperandrogenism)

Hirsutism encompasses a broad spectrum of conditions, prominently including those associated with excess androgen production or an increased response of hair follicles to androgens. Because the degree of hirsutism does not always correspond well with the amount of androgen excess, it is essential to do a thorough evaluation on women who even exhibit mild to moderate cases of hirsutism. Polycystic ovary syndrome stands out as the primary cause, being the most prevalent disorder of androgen excess (70–80%) [46]. However, the possibility of more serious underlying conditions, such as adrenal or ovarian tumors should not be overlooked. Non-classic congenital adrenal hyperplasia can manifest with hirsutism as well, which is a genetically inherited disorder of androgen excess and require genetic counselling. Thus, it would be appropriate to consider hirsutism as a sign/symptom rather than a disease and initiating a treatment without investigating the possible underlying causes would not be an advisable approach. Approach to a patient with hirsutism has been shown in Fig. 2.

### Polycystic ovary syndrome (PCOS)

The most prevalent cause of hirsutism is PCOS and accounts for 72%–82% of cases and 4%–12% of women of reproductive age [47]. Although the primary cause of the disease is an abnormality mainly in the ovaries, obesity and some environmental factors contribute to the signs and symptoms of the disease. PCOS has a multifactorial origin and it is well recognized that obesity exacerbates hyperandrogenism and menstrual dysfunction, while weight loss leads to significant improvements in terms of reproductive and metabolic aspects of the disease. In most of the cases, metabolic and reproductive abnormalities are mediated by insulin resistance and hyperinsulinemia in women with PCOS. Although it is dependent on the method used, the prevalence of insulin resistance in PCOS women is 60–70% [48]. Lean women with PCOS also have insulin resistance, however, obesity further increases insulin resistance in these patients.

Insulin resistance is defined as impaired effect of insulin in insulin sensitive tissues and predominantly manifests as inability of glucose to enter cells across cell membranes via specific glucose transporters. There are several receptor and post-receptor mechanisms in the insulin resistance of women with PCOS. Post-receptor abnormalities disrupt signal transmission downstream from the insulin receptor, selectively impairing the phosphoinositide 3-kinase pathway and leading to compromised metabolic effects of insulin and resultant hyperinsulinemia [50]. Additionally, decreased metabolic clearance rate of insulin is a well known mechanism contributing to hyperinsulinemia in women with PCOS [49]. Not all tissues show similar resistance to insulin effects and while tissues such as muscle and adipose tissue show insulin resistance some tissues like ovaries and vasculature keep their vulnerability to insulin effects. It has been shown that insulin is able to act with LH within ovarian theca cells leading to increased production of androgens. Additionally, insulin resistance mediated hyperinsulinemia promotes the arrest of pre-antral follicle development [50].

The first description of the syndrome goes to 1935 and during the last 25 years various scientific communities have published diagnostic criteria for PCOS. Currently, the most widely accepted criteria for the diagnosis of PCOS is Rotterdam Criteria which consists of: (1) oligo- anovulation, (2) clinical and or biochemical signs of hyperandrogenism, and (3) polycystic ovaries on ultrasound. The diagnosis is established when at least two of these three criteria are positive and other causes of hyperandrogenism are excluded [51]. Accordingly, PCOS can be categorized into four different phenotypes where phenotypes A and B have more pronounced insulin resistance and phenotypes C and D have less insulin resistance.

A) Classic type with androgen excess + ovulatory dysfunction + polycystic ovarian morphology.
B) Androgen excess + ovulatory dysfunction.
C) Androgen excess + polycystic ovarian morphology.
D) Ovulatory dysfunction + polycystic ovarian morphology.

In 2023, these criteria have been modified, particularly for adolescent girls [52]. PCOS can be diagnosed in adolescent females who are more than two years post-menarche, based on ovulatory failure and clinical/biochemical hyperandrogenism, after other medical conditions have been ruled out. Girls who exhibit PCOS-typical symptoms but do not fit the criteria may be categorized as “at risk for PCOS.” It is not advised to use pelvic ultrasonography to diagnose PCOS until eight years after menarche, because of multi-follicular nature of ovaries in this period.

Anti-mullerian hormone levels correlate well with polycystic ovarian morphology and now recommended in recent international guidelines for the diagnosis of adult PCOS patients. However, AMH levels change with age, there is not clear cut-off levels used for the diagnosing polycystic ovarian changes, reference values vary across assays, and AMH measurement in adolescence is not recommended owing to its low specificity [52].

In PCOS, hyperandrogenism is primarily of gonadotropin-dependent ovarian origin, with adrenal androgens contributing to a lesser degree [53]. Ovarian androgens are excessively produced in theca cells due to altered steroidogenesis and external factors such as hyperinsulinemia. It has been shown that insulin behaves like LH and augments ovarian androgen production. Patients with PCOS are at increased risk for metabolic syndrome, hypertension, hyperlipidemia, type 2 diabetes, and possibly coronary artery disease [54]. Therefore, before initiating treatment for hirsutism, it is important to diagnose PCOS if present, inform the patient about these risks, and ensure proper monitoring.

### Idiopathic hyperandrogenemia

Hyperandrogenism refers to the clinical syndrome characterized by signs and symptoms of excess androgen activity, while hyperandrogenemia specifically denotes elevated levels of androgens in the blood, which may or may not manifest clinically. Idiopathic hyperandrogenemia pertains to a subset of women exhibiting hyperandrogenism without ovarian or adrenal androgen overproduction and in the absence of specific causes like PCOS or congenital adrenal hyperplasia [55]. In a study conducted on the Turkish population, the prevalence of idiopathic hyperandrogenemia was found to be 17.4% [56]. These patients have elevated levels of one or more serum androgens, possesses normal ovulatory cycles and normal ovarian morphology. The pathophysiology remains inadequately understood; however it has been suggested that the adrenal glands may be the main source of androgens. As seen in PCOS, an increased prevalence of glucose intolerance has also been reported in these patients [55].

### Idiopathic hirsutism

Patients presenting with hirsutism, and normal serum androgen concentrations without any other pathological findings, are categorized within this group [6]. It represents 5%–15% of all cases of hirsutism and is diagnosed by ruling out other etiologies [3]. The factors underlying idiopathic hirsutism have been suggested to include a possible increase in the conversion of testosterone into DHT and/or an increased sensitivity to androgen receptors [6]. Existing data indicates that idiopathic hirsutism is more commonly observed in eumenorrheic women with mild hirsutism (mFG score between 8 and 15 for Caucasian). In contrast, women with severe hirsutism and/or other signs of hyperandrogenism such as acne or hair loss usually exhibit elevated serum androgen levels and do not fall into this category [15, 57].

It has been shown that idiopathic hirsutism and insulin resistance (IR) are associated [58]. Unluhizarci et al. demonstrated that higher basal insulin, higher homeostasis model assessment (HOMA) scores and lower glucose disappearance rates are present in patients with idiopathic hirsutism compared to controls. Accordingly, insulin resistance was prevalent in obese patients with idiopathic hirsutism, with 75% showing impaired glucose tolerance (IGT) [59]. Evidence from a large number of Korean volunteers shown that, even after accounting for biochemical hyperandrogenism parameters, insulin resistance (HOMA-IR) was strongly correlated with the FG score [60].

It is clear that any form of increased androgen level or increased androgenetic activity is necessary for hirsutism and patients with idiopathic hirsutism may exhibit relative hyperandrogenemia at the tissue level despite having serum androgens within the reference range. In other words, these patients may have higher serum androgen levels at the tissue level and previously, it has been shown that these patients have lower estradiol/testosterone ratio which is an indicator of aromatase activity [59]. In addition to relative hyperandrogenemia it has been shown that, compared to healthy women, patients with idiopathic hirsutism exhibited higher expression of 17-beta hydroxysteroid dehydrogenase and steroid sulphatase mRNA both in the arm skin and subumbilical region, which contributes to local androgen production. The authors suggest that at least in some patients with idiopathic hirsutism although the ovaries and the adrenal glands do not secrete increased amount of androgens leading to normoandrogenemia in the circulation, pilosebaceous unit locally produce increased amount of androgens contributing to hirsutism [61].

In women with idiopathic hirsutism, weight gain may exacerbate insulin resistance, potentially leading to a more complex disorder mimicking the manifestation of PCOS. This progression may occur as increased adiposity exacerbates insulin resistance, which in turn stimulates ovarian androgen production and reduces SHBG levels, elevating free androgen levels and contributing to the clinical and biochemical presentation of PCOS [47]. The above-mentioned evidence regarding insulin resistance and hirsutism might suggest that idiopathic hirsutism could potentially be an early phase of PCOS. This relationship warrants further investigation to determine if women with idiopathic hirsutism, particularly those who experience weight gain and subsequent worsening of insulin resistance, may progress to develop the more definitive features of PCOS. Such a progression would underscore the importance of monitoring and managing metabolic factors in patients with idiopathic hirsutism to prevent potential development of PCOS.

Several researchers have proposed that levels of 3α-androstanediol glucuronide (3α-diol-G), a metabolite of dihydrotestosterone (DHT), could be indicative of peripheral 5α-reductase activity [62, 63]. However, the serum concentration of this metabolite is influenced not only by the enzymatic activity in the skin but also by the circulating levels of androgen precursors [64]. As a result, elevated levels of 3α-diol-G have been observed in hirsute women with PCOS as well as in those with excess adrenal or ovarian androgens [65].

### Nonclassic congenital adrenal hyperplasia

A less common but significant cause of hirsutism in some populations is NCAH, which is caused by defects in either 21-hydroxylase, 11β- hydroxylase or 3β-hydroxysteroid dehydrogenase (3β-HSD) activity. The clinical presentation of PCOS and NCAH is quite similar including hyperandrogenemia, hirsutism, acne and oligo-anovulation. Majority of NCAH patients who seek medical care are of the 21-OH deficient form, which is linked to CYP21A2 mutations [66]. Hyperandrogenism affects about 4–10% of women worldwide and it is estimated that NCAH affects between 1% and 10% of hyperandrogenic women and 1:100 to 1:1000 of women in general population; being most prevalent in Ashkenazi Jewish and certain Middle Eastern and Indian sub-continent populations [67]. While 21-hydroxylase deficiency is the leading cause of NCAH, some populations exhibit a higher prevalence of 11β-hydroxylase deficiency. In a study conducted on Turkish population the prevalence of 11β-hydroxylase deficiency was found to be 6.5% among hirsute women [68]. A study among 78 Saudi children with CAH, 20 children (from 11 families) had 11βhydroxylase deficiency (26%) [69].

The pathogenic variants leading to 21-hydroxylase deficient NCAH typically have at least 50% residual enzyme activity and do not severely impair cortisol synthesis but result in adrenal androgen overproduction. Females with NCAH typically present with hirsutism during adolescence, sometimes accompanied by menstrual irregularities or primary amenorrhea, and nearly half of these women exhibit polycystic ovarian morphology [70]. Follicular phase serum 17-OHP levels lower than 1.7- 2 ng/ml excludes NCAH in 97% of the cases. Patients suspected for 21-hdroxylase deficiency should be evaluated with ACTH stimulation test and basal or stimulated serum 17-OHP exceeding 10 ng/ml suggest NCAH [71]. The presentation and symptoms may resemble those of PCOS, however distinguishing these conditions is essential for providing appropriate genetic counseling to patients. Another important aspect of 21-hdroxylase deficiency is heterozygous carriers. Admoni et al. showed that being a carrier is not an innocent situation and some patients with even mild mutations in one allele of 21 hydroxylase gene show symptoms of androgen excess [72].

### Insulin resistance syndromes

Insulin resistance may be inherited or acquired and may associate with acanthosis nigricans and ovarian hyperandrogenism. The type A severe insulin resistance syndrome is usually seen in adolescent girls manifesting as hirsutism and menstrual dysfunction. This syndrome is characterized by insulin receptor or rarely post receptor defect which alters insulin signal transduction system. Type B insulin resistance syndrome is caused by insulin antibodies against insulin receptor and these patients show similar clinical picture to Type A syndrome patients such as acanthosis nigricans, hirsutism and oligomenorrhea [73]. Hyperandrogenemia-insulin resistance-acanthosis nigricans (HAIR-AN) syndrome may be an underlying cause of hirsutism in a subset of patients [74].

### Androgen secreting tumors

Excluding androgen secreting tumor is the most important issue when faced with a patient with hirsutism. Compared to the commonly encountered causes of hirsutism, hyperandrogenism originating from androgen-secreting tumors is characterized by a later onset in life, exhibits rapid progression, and manifests with a severe clinical presentation. Nearly 0.2% of hyperandrogenic women have androgen-secreting tumors, of which more than half are malignant [75]. Tumors may be ovarian or adrenal in origin, with ovarian tumors typically presenting with markedly elevated testosterone levels (> 1,5 to 2 ng/mL), while adrenal-derived tumors generally exhibit significantly increased DHEAS levels (> 700 to 800 µg/dL) [76, 77]. However, there is no any androgen level discriminating between benign or malign etiologies of hyperandrogenemia. In the clinical suspicion of ovarian or adrenal tumors, low-dose dexamethasone suppression test (LDDST) for 48 h may be used. It has been suggested that lack of normalization or testosterone suppression more than 40% during the LDDST is associated with 88% specificity and 100% sensitivity in distinguishing patients with androgen secreting tumors from patients with nontumorous hyperandrogenemia [58, 75]. On the other hand, androgen secreting adrenal tumors usually associated with Cushingoid features and can be diagnosed with adrenal mass lesions. Some ovarian tumors may be too small and cannot be shown by imaging techniques and ovarian venous sampling may be necessary in these patients.

**Table 2:** Causes of hirsutism and their distinctive characteristics

Prevalence	PCOS	Idiopathic Hyperandrogenemia	Idiopathic Hirsutism	NCAH	Androgen Secreting Tumors
	70–80%	15–17%	10%	3–4%	< 1%
Menstruation	Oligomenorrhea / Normal	Normal	Normal	Oligomenorrhea / Normal	Oligomenorrhea / Normal
Serum androgens	Normal / elevated	Elevated	Normal	Normal / slightly elevated	Remarkably elevated
PCOM	+ / -	-	-	+ / -	+ / -
Insulin resistance	+ / -	-	+ / -	+ / -	-
Other distinctive features	AN* Central obesity	-	-	17-OHP ↑ Family history Ethnic predisposition	Recent onset Rapid progression Virilization

*: Acanthosis Nigricans

### Chrousos syndrome

It is also known as glucocorticoid resistance syndrome arising from a defect in glucocorticoid receptors. Compensated hyperactivity of HPA axis result is overactivation of mineralocorticoid synthesis pathway and in women also overactivation of adrenal androgen biosynthesis pathway. These patients may exhibit various degrees of hyperandrogenic symptoms in the presence of high serum cortisol and ACTH levels without Cushingoid features [78].

### Other causes of hirsutism

Despite being comparatively less common than the previously detailed etiologies, some other conditions may also cause hirsutism. Endocrinologic problems like hyperprolactinemia, thyroid abnormalities, acromegaly, and Cushing's syndrome may be linked to hirsutism, however they rarely manifest with isolated hirsutism. Certain drugs, particularly cyclosporine, danazol, diazoxide, glucocorticoids, minoxidil, testosterone, and other anabolic steroids, can result in iatrogenic hirsutism.

Distinguishing among the various etiologies of hirsutism is critical for guiding both investigation and treatment. Table 2 compares the major causes of hirsutism with respect to prevalence, menstrual pattern, androgen profile, and additional distinguishing features. Of note, while PCOS and idiopathic hirsutism account for the majority of cases, the rapid onset and pronounced androgen elevation seen in androgen-secreting tumors should always prompt urgent evaluation, even though they represent less than 1% of cases.

## Drugs used in the treatment of hirsutism

The management of hirsutism requires a comprehensive approach that balances cosmetic concerns with underlying endocrine and metabolic health. Many patients with the complaint of unwanted hair growth come to the clinic having already applied any epilation method. Although mechanical hair removal methods are among the treatment options, the first-line treatment for hirsutism is considered as medical treatment for most of the patients, as recommended in international guidelines [15]. The objectives of hirsutism management are to treat the underlying cause if possible, avoid and/or treat any potential metabolic problems that may be related, and alleviate the hirsutism and reproductive concerns [79].

Prior to initiating therapy, it is crucial to adjust the patient’s anticipations regarding the outcomes of treatment, as this enhances the probability of the patient’s adherence to the treatment regimen. It should be clearly stated to the patient that since the condition is chronic, the treatment is not curative but a long-term treatment, the results of the treatment cannot be seen before six months, and that the patient must be followed by a physician during the treatment.

### Combined estrogen-progestin oral contraceptives (COC)

Despite not having FDA approval for the treatment of hirsutism, COC are one of the most often used off-label medications for the condition. They are recommended as the first-line treatment for hirsutism by many experts in the field [15, 16]. Most of the patients also prefer these preparations because they offer non-hirsutism-related benefits like cycle control, contraception, and a decrease in other hyperandrogenic symptoms like acne or oily skin.

#### Mechanism of action

The main mechanism of action of COCs on hirsutism and hyperandrogenism is reduction of gonadotropin secretion [80]. While both FSH and LH levels are decreased by COC, decrease in LH levels are more prominent. As a result, there is a decrease in androgen release from the ovaries [81]. Another significant effect of COCs is stimulation of the hepatic synthesis of SHBG, which increases bound androgen ratio and lowers serum concentrations of free androgens [82]. Since patients with hyperandrogenism frequently have lower SHBG levels due to insulin resistance and increased androgen levels, this effect provides additional benefit for these patients [83]. All these effects act synergistically, ultimately resulting in combined oral contraceptives reducing free testosterone levels by nearly 50% [84].

#### Choice of Drug

Considering the reduced potential for suppressing ovarian androgens in newer generation COCs that incorporate *17-β-estradiol* or *estradiol valerate* with potent progestins, it is advisable to opt for COCs that include ***ethinyl estradiol (EE)***. Although EE doses such as 20–35 µg have lower risk of pulmonary embolism, myocardial infarction and stroke, increased hepatic protein synthesis leads to hypercoagulability. Many progestins are derived from 19-nortestosterone, thus they exhibit androgenic effects to varying degrees [85] The progestins *norgestimate*, *desogestrel*, and *gestodene* are examples of low androgenicity; *norethindrone* is an example of a medium androgenicity; and *levonorgestrel* and *norgestrel* are examples of comparatively high androgenicity progestins [15]. Commonly used progestins and their properties are summarized in Table 3. Drospirenone is structurally related to spironolactone and act as a weak androgen receptor antagonist. Cyproterone acetate, another progestin that is not related to testosterone structurally, exhibits antiandrogenic effects as well. Although combined oral contraceptives containing these two progestins appear ideal in terms of their additional antiandrogenic efficacy, in reality, their antiandrogenic activities seem to be below therapeutic doses. In bioassays 2 mg of cyproterone acetate was equivalent to approximately 50 mg of spironolactone and 3 mg of Drospirenone was equivalent to only 9 to 10 mg of spironolactone in terms of antiandrogenic activity. For context, the therapeutic dose of spironolactone for hirsutism ranges from 100 to 200 mg [86]. A meta-analysis concluded that COCs containing cyproterone acetate (CPA) and drospirenone do not have clinically significant advantages [15]. Regarding the dose of ethinyl estradiol, a meta-analysis of 42 studies found that suppression of serum total and free testosterone concentrations was similar between doses of 20 µg and 30 to 35 µg [87]. Both, Androgen Excess and PCOS Society and Endocrine Society clinical practice guidelines recommend initial therapy with an OC containing the lowest effective dose of ethinyl estradiol (usually 20 µg) and a low-risk progestin [15, 16]. It has recently been shown that although EE/CPA is the most effective treatment on hirsutism compared to other COCs with other progestins, it is associated with more thromboembolic events [88].

Overall, COCs are commonly used particulary in patients with higher FG score and also in women with severe menstrual disturbances. In patients with severe hirsutism associated with benign conditions, COCs may be combined with some anti-androgens such as finasteride or spironolactone resulting in earlier response in terms of hirsutism.

**Table 3:** Commonly used progestins and their properties [15, 89]

Progestin/Dose	Generation	Androgenic effect	Relative VTE Risk
Norethindrone 0.5–1.0 mg	1	Medium	2.6
Levonorgestrel 0.15 mg	2	High	2.4
Norgestimate 0.25 mg	2–3	Low	2.5
Gestodene 0.075 mg	3	Low	3.6
Desogestrel 0.15 mg	3	Low	4.3
Drospirenone 3 mg	4	Antiandrogen	4.1
Cyproterone acetate 2 mg	—	Antiandrogen	4.3

### Anti androgens

Although antiandrogens may not be the first-line treatment for hirsutism due to various reasons, they are notably potent medications. The antiandrogens finasteride, flutamide, and spironolactone all significantly reduced hirsutism ratings in a network meta-analysis of pharmacologic treatments for hirsutism [90]. Each of the three medications had comparable effects. In strategies that involve the use of multiple antiandrogens, the concurrent administration of spironolactone and finasteride demonstrated greater efficacy compared to the use of spironolactone alone [91]. It is important to.

acknowledge that antiandrogens may lead to severe developmental complications in male fetuses, thus necessitating to ensure contraception while undergoing treatment with these agents. Although COCs are considered the first drug in the treatment of hirsutism, particularly in women with PCOS, we think that anti-androgens may be preferred alone in patients with relatively mild hirsutism without any menstrual disturbances. There are four well-known antiandrogens that have been studied for their effects on hirsutism:

#### Spironolactone

Spironolactone, an aldosterone antagonist, exhibits a dose-dependent competitive inhibition effect on androgen receptors, while also demonstrating a mild inhibitory effect on 5α-reductase and certain other steroidogenesis enzymes [92]. Spironolactone is generally considered safe in terms of side effects, making it the most commonly preferred antiandrogen. The most commonly seen side effects are dizziness, breast tenderness, headache, polymenorrhea and transient polyuria. It can be administered in a daily dose of 100 mg. It can be used alone or in combination with other antiandrogens or with COCs [93].

#### Drospirenone

It is a progestin derived from 17-alpha spironolactone having anti-androgenic and anti-mineralocorticoid effects. Drospirenone display antiandrogenic activity by competitive binding to the androgen receptor. The drug also inhibits ovarian androgen production [94]. It has been used in combination with an estrogen rather than being used alone. However, recently, it has been shown that drospirenone-only pills may be used in patients with PCOS who cannot take estrogen [95].

#### Cyproterone acetate

Cyproterone acetate is a progestogenic agent exhibiting anti-androgenic properties, exerting its influence through competition with DHT for androgen receptor binding [96]. It further contributes to the reduction of serum gonadotropin and androgen concentrations. It may be used in a minimal dosage (2 mg) as the progestin element in combined oral contraceptives (COCs), or in higher dosages (12.5 to 100 mg) as a single treatment or in combination with estrogen. However, it has been shown that various low or high dosages of CPA did not differ in decreasing hirsutism scores. CPA has been associated with rarely seen liver toxicity, irregular menstrual bleeding, nausea, decreased libido, and depression [97].

#### Finasteride

Finasteride inhibits type 2 5α-reductase activity, leading to a reduction in DHT production. Since the increased activity of 5α-reductase in hirsutism likely involves both type 1 and type 2 enzymes, finasteride may only exert a partial inhibitory effect. Due to its very low side effect profile finasteride is a good therapeutic choice for the treatment of hirsutism and it is effective in 5 mg dose per day [98]. **Dutasteride**, approved for treating prostate cancer in men, inhibits both type 1 and type 2 isoenzymes. Despite its potential appeal as a treatment option for hirsutism, there is yet to be clinical evidence to endorse its application in this context [99].

#### Flutamide

Flutamide, a nonsteroidal antagonist of androgen receptors, is primarily used in treating prostate cancer. However, it has also been utilized off-label for the management of hirsutism. Flutamide shows its effect on the androgen receptor and various doses have been used in the management of hirsutism [98, 100]. Lower doses of flutamide than doses used for prostate cancer have shown no hepatotoxic effects, however, flutamide is not recommended routinely in current hirsutism treatment [15, 101]. Flutamide licensing guidelines suggest checking serum transaminase levels prior to starting treatment, monitoring them monthly during the first four months, and then annually. The medication should not be used in patients whose transaminase levels are more than twice the normal upper limit [102].

Considering all this information, the use of antiandrogens is deemed suitable in the following circumstances:

1. As an adjunct to oral contraceptives in cases of severe hirsutism,
2. When an adequate response to oral contraceptives is not achieved at the six-month check-up,
3. In women who have regular menstrual cycles, for whom oral contraceptives are contraindicated or who prefer not to use oral contraceptives, in conjunction with a contraceptive method.

### Other medical treatments

#### Metformin

**Fig. 3:**
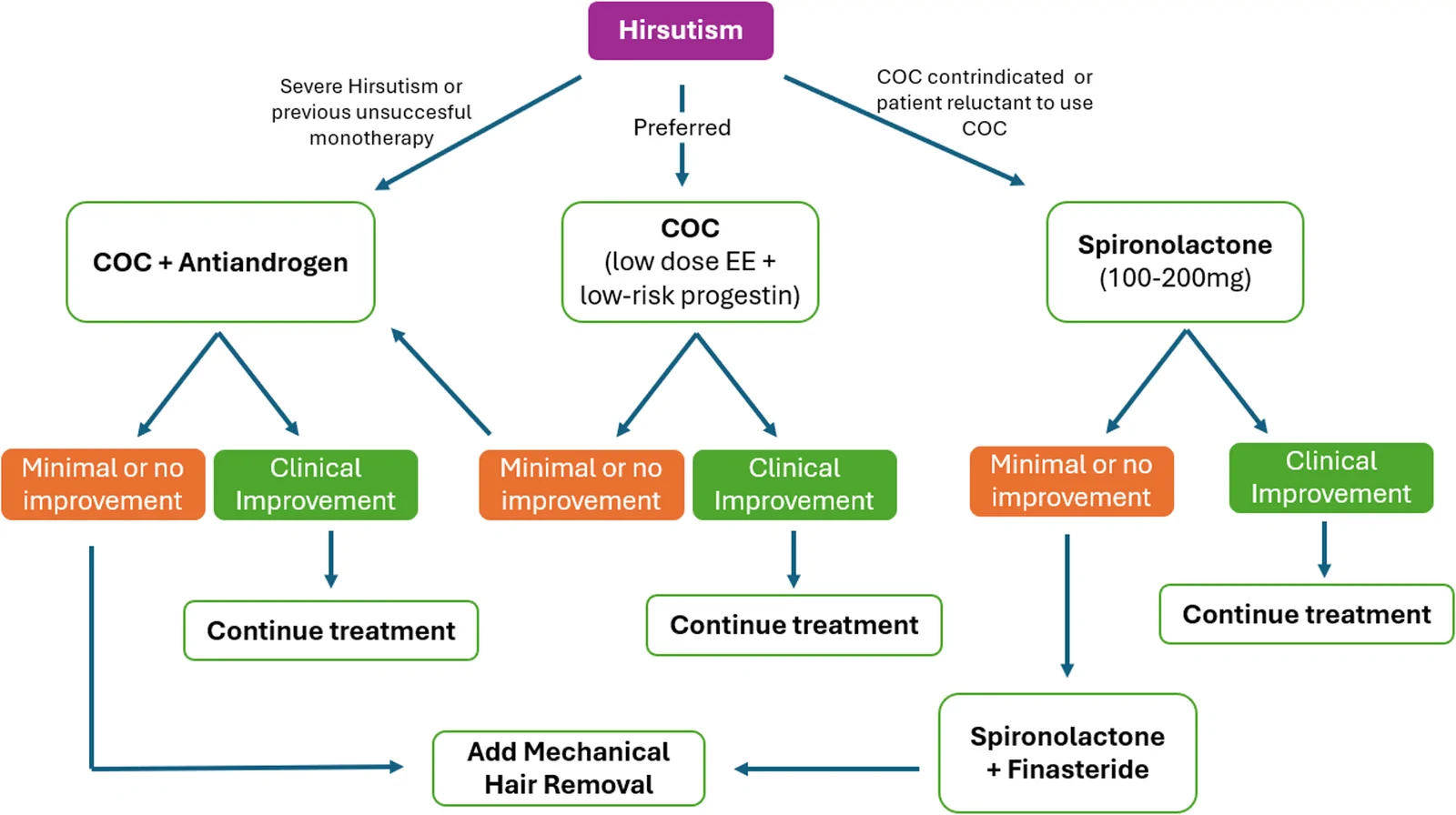
Treatment algorithm for hirsutism (Mechanical hair removal may be added in all steps of treatment. Metformin and other insulin sensitizers may be added to any treatment option in case of diagnosed insulin resistance and/or metabolic abnormalities)

Although most of the androgen excess disorders are associated with insulin resistance, PCOS is the important one from this point of view [61]. Insulin resistance and hyperandrogenemia are strictly related issues questioning which came first – the chicken or the egg? Thus insulin sensitizing agents, mainly metformin, has been considered in the management of patients with hirsutism for diverse beneficial effects. Figure 3 provides a treatment algorithm for hirsutism.

Metformin have the potential to enhance various clinical metrics in PCOS, yet as of now, there is lack of adequate evidence to confirm the efficacy of this methodology in the treatment of hirsutism. On the other hand, since high insulin levels increase LH mediated ovarian androgen secretion, combination of metformin and COCs will aid improvements in several mechanisms particularly in women with PCOS. Some clinical trials have shown that metformin alone as well can produce modest but statistically significant improvements in hirsutism: for example in a double-blind, placebo-controlled crossover study Kelly et al. observed a reduction in FG score, decreased hair growth velocity, weight loss, and modest improvements in SHBG and free androgen index after metformin treatment relative to placebo in women with PCOS [103]. Also, in a prospective study of 40 obese PCOS women treated with metformin 500 mg three times daily for 8 weeks, Kazerooni et al. reported a significant decline in total testosterone, fasting blood sugar, BMI, acne, and hirsutism score [104]. However, a recent meta-analysis comparing COC, metformin, and COC+ metformin concluded that for hirsutism scores, there was no significant difference between metformin alone versus COC, nor between COC alone versus the combined treatment; however biochemical androgen markers (e.g. free androgen index, SHBG, total testosterone) and metabolic parameters (insulin, triglycerides) showed greater improvement in COC + metformin compared to COC alone or metformin alone [105]. Thus, while metformin may not match COCs in terms of reducing visible hair growth speed or extent (clinical F-G score improvements), it contributes to reducing androgen levels, improving metabolic measures (insulin resistance, hyperinsulinemia) and may have additive effects when combined with COCs in selected patients.

**Gonadotropin-releasing hormone analogs** can mimic the effects of medical oophorectomy to manage persistent hirsutism caused by ovarian hyperandrogenism. It has been used rarely in common disorders associated with hirsutism such as idiopathic hirsutism, PCOS or idiopathic hyperandrogenemia. The resulting hypoestrogenic side effects often require the supplementation with an oral contraceptive pill or the addition of estrogen/progestin therapy [106]. Postmenopausal women with ovarian hyperandrogenism due to benign ovarian tumors may also benefit from GnRH analogue therapy particularly in patients with high operative risk or who are reluctant to surgery. Besides their use in the treatment of some forms of ovarian hyperandrogenism, GnRH analogue has been used to confirm gonadotropin-dependent ovarian hyperandrogenism. This test relies on serum testosterone suppression after the administration of a single dose of triptorelin (3 mg), leuprorelin (3.75 mg) intramuscularly or subcutaneously. Suppression of testosterone by more than 50% following GnRH analogue at three to four weeks of application, suggest a possible benign source of ovarian hyperandrogenism [107].

**Glucocorticoids** are capable of inhibiting adrenal androgen synthesis, mostly used in patients with congenital adrenal hyperplasia, however, the dose required for inhibiting androgen synthesis is much higher than glucocorticoid replacement dose and leads to Cushingoid symptoms in many patients. Additionally, since many patients do not exhibit glucocorticoid deficiency in daily life, patients with NCAH do not require glucocorticoids. Moreover, many patients with NCAH respond well to anti androgens and/or OCP in terms of hirsutism, therefore the most important indication for glucocorticoids in NCAH patients are inducing ovulation for pregnancy.

#### Topical Treatment

By permanently blocking ornithine decarboxylase, which catalyzes the rate-limiting step for follicular polyamine production, **eflornithine** lowers the pace of hair growth. The FDA has approved eflornithine hydrochloride cream 13.9% as a topical therapy for women’s undesired facial hair [108].

### Combination therapies

Due to the distinct mechanisms of action of above mentioned drugs, combining two drugs can enhance the therapeutic effect achieved. This approach leverages the synergistic potential of the medications, potentially leading to improved clinical outcomes compared to monotherapy. With this rationale, numerous combination therapies have been studied.

**COC and antiandrogen** combination therapy is shown to be more effective than COC alone therapy in several studies. A study compared the clinical efficacy and safety of a COC (cyproterone acetate, 2 mg, and ethinyloestradiol, 35 µg) combined with **spironolactone** (100 mg) versus COC alone in treating hirsutism in 50 women. The results showed that while both groups experienced a significant reduction in hirsutism scores after one year, the combination of COC plus spironolactone led to a greater reduction in hirsutism scores compared to COC alone [109]. A more recent study which was conducted on 200 patients with PCOS revealed that the efficacy of suppressive therapy for hirsutism was greater with COC + spironolactone than with either drug alone [110]. The authors noted that combination therapy not only led to better objective outcomes but also resulted in higher patient satisfaction compared to monotherapy. Additionally, they observed that patient satisfaction increased as the duration of treatment extended.

Studies which compared COC (cyproterone acetate, 2 mg, and ethinyloestradiol, 35 µg) and **finasteride** combination therapy with COC alone reported that the percentage change in the Ferriman–Gallwey score from baseline was higher in patients treated with COC plus finasteride compared to those treated with COC alone. This suggests that adding finasteride to COC has a synergistic effect, leading to a more significant reduction in hirsutism scores [111, 112].

In approaches that combine multiple antiandrogens, the combination of **spironolactone with finasteride** proved to be more effective than spironolactone alone in two studies conducted in women with PCOS or idiopathic hirsutism [91, 93]. According to these two studies, antiandrogen combination therapy seems to be both effective and safe in treating hirsutism, as far as contraception is ensured. The authors concluded that using antiandrogen drugs with different mechanisms of action together might serve as an alternative therapeutic approach for managing hirsutism.

However, not all the combinations have additive effect in the management of hirsutism although theoretically seems logical. An example for this combination is finasteride plus flutamide, where flutamide alone has similar effects to combination therapy in the management of hirsutism [98].

In a comparative analysis between ethinyl estradiol plus cyproterone acetate with or without **metformin** therapy in the treatment of PCOS induced hirsutism, metformin was found to be only marginally effective in treating hirsutism, but was highly effective in regulating menstrual cycle and decreasing obesity [113]. In another study on effectiveness of low-dose ethinylestradiol/cyproterone acetate and ethinylestradiol/desogestrel with and without metformin on hirsutism in PCOS, there was no added benefit of metformin in terms of hirsutism [114].

### Mechanical hair removal

#### Temporary Methods

Depilation, like shaving, doesn't affect hair follicle biology or change hair characteristics but makes regrown hair feel thicker. Methods such as plucking and waxing are cost-effective but may cause discomfort and potential side effects like scarring and hyperpigmentation. Chemical depilatories dissolve hair by breaking disulfide bonds, they may lead to possible irritant dermatitis and hyperpigmentation.

#### Permanent Methods

**Electrolysis**, a century-old hair reduction method, remains under-researched. It involves directing electrical current through a wire into hair follicles, with galvanic electrolysis technique causing toxic reactions and thermolysis technique generating heat for hair removal. A combination, “The Blend,” is deemed more effective [115]. Despite pain associated with thermolysis and blend techniques, electrolysis is seen as effective for permanent hair reduction, with topical anesthetics reducing discomfort.

**Photoepilation** has been introduced less than two decades ago and has rapidly ascended to become the third most common nonsurgical aesthetic procedure in the United States, following botulinum toxin and hyaluronic acid injections. This technique, employing light pulses absorbed by melanin in the hair shaft and follicle, achieves selective photothermolysis of pigmented terminal hair follicles, utilizing various lasers (ruby, alexandrite, diode, Nd: YAG) and Intense Pulsed Light (IPL) sources [116]. Adjustments in fluence and pulse duration cater to individual patient hair and skin types, aiming for irreversible thermal damage to hair follicles without harming surrounding skin. The FDA has also cleared home-use diode laser IPLs for over-the-counter sale, though their efficacy for permanent hair reduction may vary.

Even though these methods are effective, in women who have underlying hyperandrogenism, the constant stimulation of hair follicles by endogenous androgens can lead to hair regrowth. The suppression of endogenous androgens through pharmacological means can avert this recurrence.

## Limitations

As a narrative review, this work is subject to inherent limitations including the absence of a formal systematic search protocol and lack of risk-of-bias assessment. In addition, the heterogeneity of study populations and diagnostic criteria across the included studies limits the comparability of treatment outcomes, particularly across different ethnic groups.

## Conclusion and future directions

Hirsutism is a multifactorial clinical disease that lies at the intersection of endocrinology, dermatology, and reproductive medicine. While androgen excess still acts as the core pathophysiologic mechanism, heterogeneity of the underlying etiology ranging from PCOS and nonclassical congenital adrenal hyperplasia to idiopathic hirsutism imposes the requirement for tailored diagnostic and treatment approaches. Discordance between clinical disease severity and levels of circulating androgens highlights the importance of local androgen metabolism, receptor sensitivity, and gene polymorphisms in the pilosebaceous unit. Since hirsutism is a symptom rather than a disease itself, identifying the underlying etiology before treatment is essential not only to guide appropriate therapy but also to detect potentially serious endocrine or neoplastic disorders that may initially present with excessive hair growth. Combined oral contraceptives should be the first drug in patients with severe hirsutism and also in patients with oligo-amenorrhea. On the other hand, anti-androgens may be the first drug in patients with idiopathic hirsutism. In some patients combining COCs and anti-androgens may be more effective than either drug alone. During the course of the disease, combination therapy may be changed to COC or anti-androgen alone therapy. Patients with regular cycles may be treated with spironolactone or finasteride or the combination of both drugs in patients with mild to moderate hirsutism. In patients with mild hirsutism and in patients who have contraindications for COCs are good candidates for an anti-androgen alone therapy.

Future research should focus on local molecular mechanisms, the role of insulin resistance in tissue-specific hyperandrogenism, and biomarkers that identify patients likely to respond to treatment. Clinically, treatment should include a combination of medical and cosmetic measures and a treatment of the psychosocial consequences that have a major impact on quality of life. Patient-oriented, multidisciplinary care and continued refinements in diagnostic thresholds among various ethnic groups will further individualize care and enhance outcome in women affected by hirsutism.

## Data Availability

No datasets were generated or analysed during the current study.
